# Development and Application of Endothelial Cells Derived From Pluripotent Stem Cells in Microphysiological Systems Models

**DOI:** 10.3389/fcvm.2021.625016

**Published:** 2021-02-15

**Authors:** Crystal C. Kennedy, Erin E. Brown, Nadia O. Abutaleb, George A. Truskey

**Affiliations:** ^1^University Program in Genetics and Genomics, Duke University, Durham, NC, United States; ^2^Department of Biomedical Engineering, Duke University, Durham, NC, United States

**Keywords:** induced pluripotent stem cells, cell differentiation, vascular endothelium, vascular tissue engineering, organoids

## Abstract

The vascular endothelium is present in all organs and blood vessels, facilitates the exchange of nutrients and waste throughout different organ systems in the body, and sets the tone for healthy vessel function. Mechanosensitive in nature, the endothelium responds to the magnitude and temporal waveform of shear stress in the vessels. Endothelial dysfunction can lead to atherosclerosis and other diseases. Modeling endothelial function and dysfunction in organ systems *in vitro*, such as the blood–brain barrier and tissue-engineered blood vessels, requires sourcing endothelial cells (ECs) for these biomedical engineering applications. It can be difficult to source primary, easily renewable ECs that possess the function or dysfunction in question. In contrast, human pluripotent stem cells (hPSCs) can be sourced from donors of interest and renewed almost indefinitely. In this review, we highlight how knowledge of vascular EC development *in vivo* is used to differentiate induced pluripotent stem cells (iPSC) into ECs. We then describe how iPSC-derived ECs are being used currently in *in vitro* models of organ function and disease and *in vivo* applications.

## Introduction

Key functions of blood vessels are to transport oxygen, nutrients, hormones, and immune cells to various tissues of the body, and to move waste products from tissues to the kidneys, liver, and intestine for metabolism and removal. Endothelial cells (ECs) line the inner walls of all blood vessels in contact with blood and regulate vessel tone, prevent thrombosis and leukocyte adhesion, and regulate the transport of fluid and solutes between blood and tissues. Arteries and arterioles dilate and constrict, redirecting blood to respond to changing metabolic demands. Immune cells travel through the blood to sites of infection or tissue damage where they adhere to activated capillary endothelium and transmigrate into the tissue to eliminate the infectious agent.

Large and medium size vessels consist of an endothelial layer in contact with blood and a medial layer with smooth muscle cells and extracellular matrix. The microvasculature, which consists of the smallest arterioles, capillaries, and venules, form a network that regulates local blood perfusion and exchange between the bloodstream and the surrounding tissues (1, 2). Capillaries contain a single layer of ECs surrounded by a basement membrane. Microvascular ECs actively regulate nutrient transport and control organ development, homeostasis, and tissue regeneration (2). Oxygen and other non-polar small molecules diffuse through most ECs into surrounding tissues and interstitial fluid. Large molecules pass across the endothelial layer by transcytosis between cells with incomplete barrier function or by endocytosis (3).

ECs respond to cytokines or bacteria *via* upregulation of genes involved in inflammation (4). For example, after exposure to TNFα or chronic low and oscillating shear stress, ECs express monocyte chemotactic protein, IL-1β, and adhesion molecules for leukocytes, such as VCAM1, E-selectin and ICAM1 (5). EC behavior also governs the microenvironment, regulating growth factors necessary in organ development and tissue regeneration and maintaining homeostasis in cases of reduced blood flow or inflammation. Alterations in endothelial function are involved in cardiovascular diseases such as atherosclerosis, hypertension, diabetes (6), and potentially, various types of dementia (7).

The mechanosensory nature of ECs allow them to respond to the forces acting upon them by blood flow through vessels (8). Laminar flow exerts a shear stress upon ECs that changes its transcriptional profile. Away from arterial branches, the shear stress waveform promotes signaling and gene expression by the vascular endothelium that inhibit thrombosis and inflammation. Conversely, in low and oscillating shear stress environments, the endothelium releases vasoconstrictors such as endothelin and promotes a pro-oxidative and inflammatory phenotype (9).

The transporters present on endothelium and the types of junctions formed between ECs vary among tissues, reflecting the specific function of the endothelium. For example, in the brain, microvascular ECs are connected with a continuous layer of tight junctions that limit the transport of solutes (2). In contrast, sinusoidal endothelium in the liver has a discontinuous basement membrane for the free movement of water and larger molecules (2).

Successful implantation and integration of engineered tissues requires rapid formation of vasculature to provide nutrients and prevent cell death (10, 11). *In vitro*, capillary networks can be used to model the microvasculature and study metastasis.

Donor-derived ECs for regenerative medicine or *in vitro* disease modeling can be obtained from adipose tissue, endothelial precursors isolated from blood, or by differentiation of induced pluripotent stem cells (iPSCs) derived from fibroblasts or blood cells. Limitations of primary cells are the finite number of cell divisions before senescence and challenges isolating tissue-specific ECs. In contrast, iPSCs remain proliferative over dozens of passages and protocols to differentiate iPSCs to various tissue-specific ECs continue to develop. An ongoing challenge is establishing the extent to which iPSC-derived ECs reflect the function of mature primary ECs.

This review provides a summary of the development of human vascular endothelium *in vivo* relevant to differentiation of iPSCs to ECs. We examine different approaches to generate ECs from iPSCs and evaluate the extent of differentiation and maturation of iPSC-derived ECs. Finally, we discuss applications of iPSC-derived ECs.

## Overview of Vascular Endothelial Development and Function

### Development From Mesoderm to Vascular Endothelium

The developing mammalian embryo consists of the ectoderm, mesoderm, and endoderm cell layers. The mesoderm layer gives rise to various cell types including ECs, smooth muscle cells (SMCs), and pericytes. ECs from the mesoderm first form an immature tubular network called the primitive vascular plexus, which matures into an orderly network of arteries, veins, and capillaries (3, 12, 13). As the heart begins to beat, blood flow through the vasculature remodels ECs to enhance their functional properties, and SMCs and pericytes are recruited to stabilize this vasculature. The formation of the lymphatic vessels completes the full development of the circulatory system. Cross-talk between ECs and neighboring tissues orders the specification of endothelium in and around specific organs, depending on their precise function for that organ system (13).

**Figure 1:**
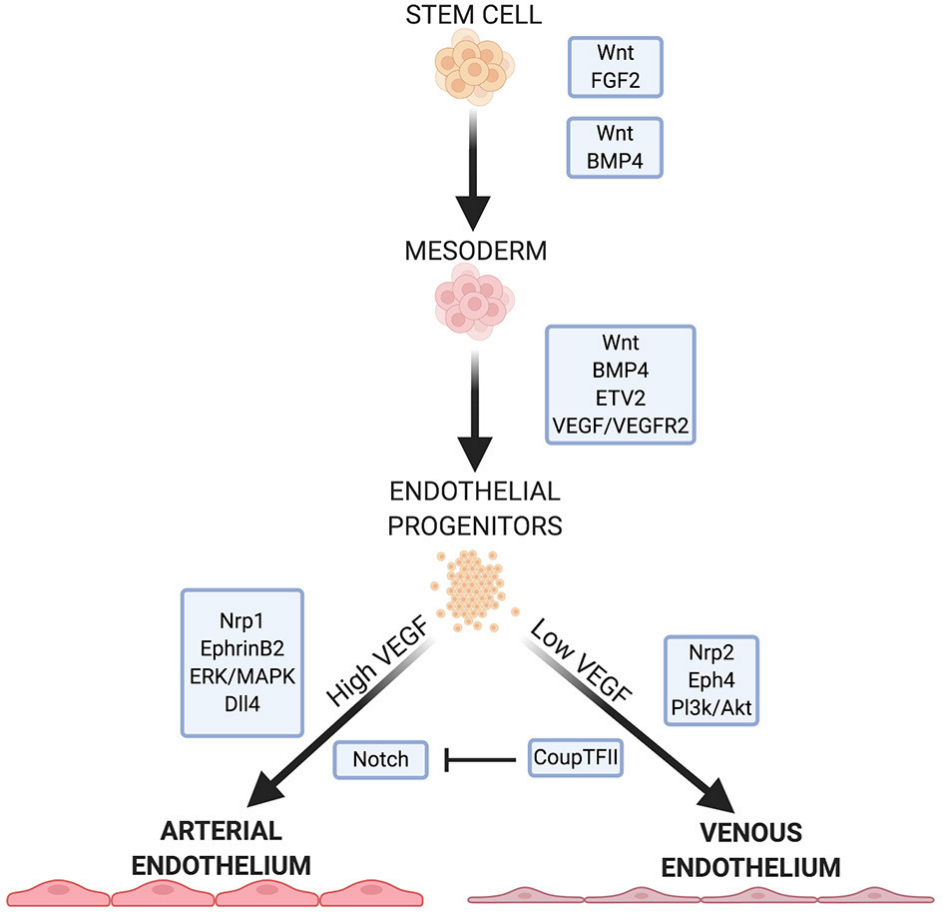
Summary of some important factors and pathways in EC development. Wnt signaling is necessary for specification of mesoderm and proper function in arterial ECs. FGF2, BMP4, and ETV2 are crucial for mesoderm formation and EC specification. VEGF/VEGFR2 signaling further specifies EC fate, with high and low concentrations leading to arterial or venous EC specificity, respectively. VEGF signaling continues to mediates downstream pathways and receptors that further direct arterial or venous EC specificity.

A vast network of signaling molecules, transcription factors, and growth factors work together to direct stem cells toward mesodermal specification and endothelial identity *in vivo* (3). Here, we will highlight some of these, focusing on pathways and molecules that are typically involved in EC differentiation protocols (Figure 1).

Wnt signaling is crucial for mesoderm formation and EC development. The canonical Wnt signaling pathway inactivates the complex that targets β-catenin for phosphorylation and subsequent degradation (14, 15). β-catenin links the cytoplasmic tails of EC-specific cadherins to the cytoskeleton. When a Wnt factor binds its receptor, β-catenin translocates to the nucleus and activates the transcription factors T-cell factor/lymphoid enhancer factor (14, 15) which regulate mesoderm formation. In addition, Wnt/β-catenin signaling is important for overall vasculogenesis in embryonic development. This is linked to Dll4/Notch signaling (discussed below), and Notch inhibition causes an increase in venous EC specification (13, 16).

Non-canonical Wnt signaling pathways may play a role in EC development. Inhibition of Wnt signaling leads to a significant decrease in mesodermal cells expressing vascular endothelial growth factor receptors (VEGFR) (17) which directs mesoderm cells to ECs. Wnt inhibition significantly downregulates ETV2 expression—a potent and transiently expressed transcription factor in EC development (17).

Fibroblast growth factor 2 (FGF2) isoforms activate multiple FGF receptors to promote cell maintenance, growth and migration (3, 18). FGF2 is necessary to maintain human embryonic stem cells (hESCs) and iPSCs in their pluripotent states by modulating downstream effectors of Wnt signaling (19, 20). While FGF2 has a role in mesoderm induction and patterning in non-human models, FGF2 does not directly promote mesoderm formation in human stem cells in culture (3, 21). Rather, FGF2 regulates angiogenesis in mature ECs, leading them to form capillaries *in vitro* (18).

Bone morphogenic protein 4 (BMP4) is part of the TGF-β superfamily of proteins and is crucial in directing human stem cells to a mesodermal lineage while promoting EC generation from the mesoderm (3, 22). BMP4 acts downstream of the endoderm-derived regulator Indian hedgehog (IHH) to initiate the formation of ECs and the vascular plexus from the mesoderm (3, 21, 22). In human cells, BMP4 acts upstream of factors responsible for arteriogenesis (3) and also initiates endodermal release of FGF2 in human stem cells (3, 21). The addition of BMP4 induces the mesoderm *in vitro* (3), and in the absence of IHH, it is sufficient to induce EC lineage (3, 21, 22).

FGF2 and IHH act on adjacent endodermal cells to produce vascular endothelial growth factor A (VEGF-A). FGF2 acts on select mesodermal cells to express VEGFR2 (also called Flk-1) (23), a receptor for VEGF in immature ECs (24). VEGF plays a role in regulating cell proliferation, vasculogenesis, and angiogenesis (24, 25). Signaling between VEGF and its receptor is important for endothelial fate, and the balance between the two also directs ECs to an arterial or vascular phenotype (discussed below). VEGF promotes the proliferation of ECs and the vasoactivity of vasculature through nitric oxide (NO) production (25). In hESCs, BMP4 is necessary for VEGFR2 expression (26), and acts, together with the Wnt and Notch pathways by activating the transcription factor ETV2, which binds to the VEGFR2 promoter (17).

VEGF binding to VEGFR2 induces p38-MAPK signaling which activates the promoter region of ETV2, a member of the ETS (E26 transformation-specific) family of transcription factors (27). ETV2 is highly expressed in the mesoderm and in developing vascular ECs but is downregulated after EC maturity (3, 28). ETV2 is important in mesoderm-to-EC development, and is necessary and sufficient for vasculogenesis (3). Many endothelial specific genes, including VEGF, contain motifs in their regulatory regions that are recognized by ETS proteins (29). ERG (ETS-related gene) regulates the expression of junction proteins such as VE-cadherin and claudin-5, which maintain the barrier function of the endothelium (29–31). ERG also mediates Dll4 expression (29), which is important for arterial specification (32).

### Arterial/Venous Specification of Vascular ECs

Due to the differing functions and environments of arteries and veins, venous and arterial ECs exhibit specific phenotypes. Before blood flow arises, arterial and venous endothelium are established by expression of EphrinB2 and the receptor EphB4, respectively (33, 34). EphrinB2-expressing angioblasts form the main arterial vessels of the vascular plexus while EphB4 positive cells assume a venous identity. Neuropilin 1 (Nrp1), a co-receptor with VEGFR2, also plays a role in ordering the formation of arteries and veins before blood flow. Progenitors near to the nerves in developing embryos express Nrp1 which increases VEGF signaling in these cells. Positive feedback leads to increased Nrp1 expression in the presence of VEGF. The increased VEGF signaling leads to arterial specification in these progenitors. Nrp1 is exclusively expressed in arterial ECs, with Nrp 2 expressed in venous ECs.

EphrinB2 is upregulated by genes which respond to the Notch pathway (33). High concentrations of VEGF lead to an upregulation of EphrinB2 and arterial specification (33), while low VEGF concentration allows upregulation of COUP-TF2, suppressing Notch, and leading to an upregulation of EphB4 and venous EC specification (2). The ERK/MAPK and Pl3k/Akt pathways, both mediated by Notch signaling, induce arterial lineage and inhibit arterial markers, respectively. Dll4, also uniquely expressed in arterial ECs, is upregulated by MAPK and downregulated by Pl3k. Dll4 is also upregulated by the arterial-specific Sox17.

Shear stress induced by flowing blood is important for vascular remodeling and EC maturity. As the heart pumps blood through the immature vascular network, fusion of vessels occurs which increases vessel diameter. Blood flow also encourages EC migration to lengthen and enlarge vessels (35). *In vivo*, adult ECs lose this plasticity that encourages cell maturity under shear stress. Applying shear stress to iPSC-derived ECs, which often show an immature phenotype, results in an upregulation of arterial markers such as EphrinB2 and Notch1 (33).

## Differentiation and Characterization of Vascular ECs from Human Stem Cells

**Figure 2:**
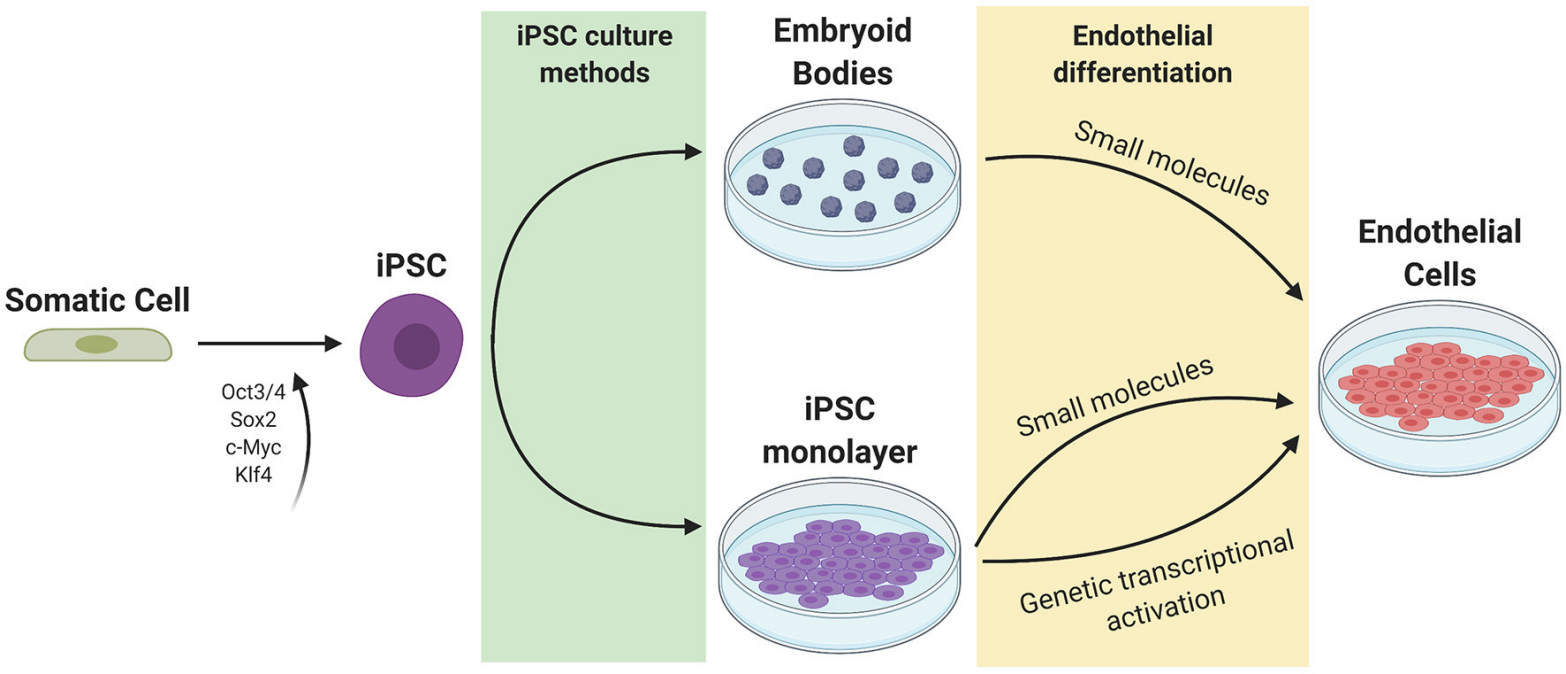
Current methods of iPSC culture and differentiation. Somatic cells are reprogrammed to iPSCs using 4 factors (Oct3/4, Sox2, c-Myc, and Klf4). iPSCs can be cultured as a monolayer or as 3D embryoid bodies. Endothelial differentiation can be achieved through the addition of small molecules to promote mesoderm then endothelial specification, or through genetic transcriptional activation or exogenous addition of ETV2.

The general approach to stem cell differentiation is to mimic developmental processes *in vitro* to achieve a desired cell fate. Stages of stem cell differentiation to ECs include lateral mesoderm induction, followed by vascular endothelial specification and selection (Figure 2). Three common approaches to EC differentiation are: (1) 3D embryoid body (EB) formation of stem cells prior to EC specification, (2) addition of key molecules to stem cells in 2D/monolayer culture, and (3) genetic manipulation or exogenous expression of key genes responsible for the signaling cascade that results in EC fate. Table 1 summarizes the EC differentiation protocols discussed below.

**Table 1:** Summary of highlighted EC differentiation protocols.

**EC differentiation approach**	**Methodology summary**	**Duration, days**	**Reference**
*Embryoid body*	EB induction: BMP4, bFGF and Activin A. Selection of cardiovascular progenitors: low KDR and negative cKIT expression. EC differentiation: ECs induced with VEGF and bFGF. EC selection: CD31	20	Lin et al. (38)
*iPSC monolayer with addition of small molecules*	Mesoderm specification: BMP4, Activin A, VEGF and CHIR Vascular specification: VEGF and TGF-β inhibitor EC selection: CD144, CD31	10	Orlova et al. (39)
	Mesoderm specification: BMP4, CHIR Vascular specification: Forskolin, VEGF EC selection: CD144	6	Patsch et al. (40)
	Epiblast induction: FGF2, RA, BMP4, CHIR Primitive streak induction: FGF2, BMP7, CHIR Mesoderm specification: BMP7 Vascular specification: VEGF, IWR1 EC selection: CD144	4	Tsujimoto et al. (41)
*iPSC monolayer with transcriptional activation*	EC specification: iPSCs transfected with ETV2 transcription factor 20–50% cells express KDR, CD31, CD34	7	Elcheva et al. (42)
	Mesoderm specification: CHIR. modRNA expressing ETV transfected into mesoderm cells. EC selection: CD144, CD31	4	Wang et al. (43)

*A summary of the discussed protocols for iPSC differentiation to endothelial cells. Three approaches are highlighted; namely embryoid body, monolayer with addition of small molecules, and transcriptional activation. Most protocols include a mesoderm specification step followed by vascular specification. Selection of successfully differentiated ECs often use CD144 (VE-cadherin) and CD31 as selection markers*.

### Embryoid Body Approach

In the EB approach, stem cells are plated as aggregates and allowed to spontaneously differentiate in a similar fashion as in embryonic development. This results in a heterogenous population of aggregated cells and therefore low efficiency of EC generation. To increase the yield of ECs derived from this method, key molecules are added to the culture medium that induce mesoderm and EC lineages. Typically, these 3D aggregates are then dissociated, and ECs are selected and plated as a monolayer for expansion (36, 37).

ECs derived from iPSCs using the EB approach exhibit a heterogenous outcome and low efficiency of EC formation. Lin et al. set out to derive cardiomyocytes, smooth muscle cells, and ECs using the EB approach to differentiation (38). EBs are first induced with BMP4, bFGF, and activin A to guide them toward a cardiovascular fate. For EC/SMC specification, EBs are treated with VEGF and bFGF from day 4. Multipotent cardiovascular progenitors are then sorted based on low KDR and negative c-kit levels, dissociated, and cultured in a monolayer at day 6. By day 8, CD31 is detected in cultured cells. Cells are treated continuously with bFGF and VEGF until day 20. FACS sorting using CD31 as a marker separated ECs from SMCs, with a yield of 20 and 70% respectively. Tube formation on Matrigel and LDL endocytosis confirmed functional properties of these iPSC EB-derived ECs.

### Addition of Small Molecules to iPSCs in 2D Monolayer

**Figure 3:**
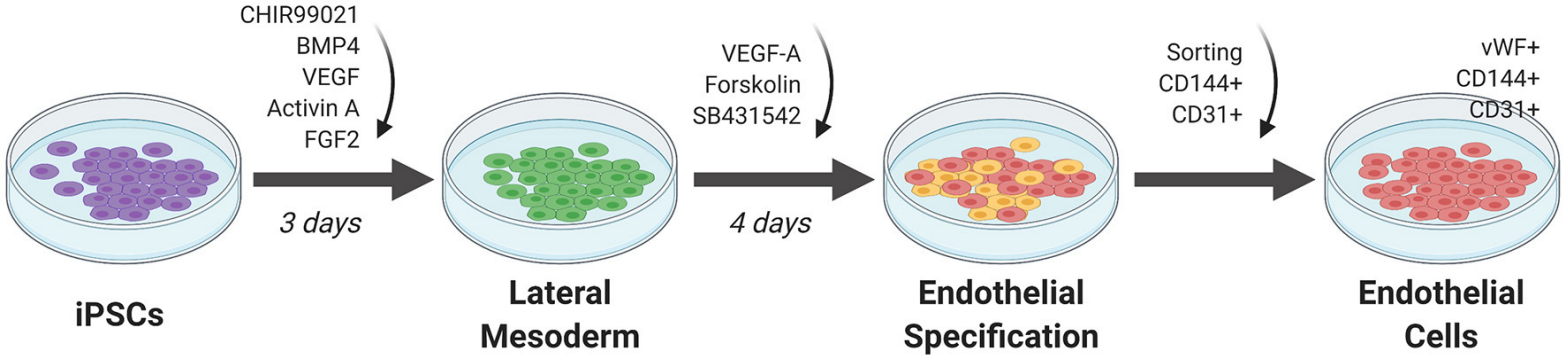
Monolayer method of EC differentiation from iPSCs using small molecules. iPSCs transition to lateral mesoderm through addition of CHIR99021, BMP4, and FGF2 for 3 days. Endothelial specification is induced for 3 to 5 days using VEGF-A, SB431542, and forskolin. Sorting for CD144^+^ and CD31^+^ cells generates a pure differentiated EC population.

Initial attempts at EC differentiation utilized 2D co-culture with murine stromal cells as a feeder layer for stem cells. This undirected differentiation approach results in a heterogenous population of cell types and low efficiency in generating ECs (36). Current approaches involve a feeder-free stem cell culture approach for EC differentiation (Figure 3). In general, stem cells are first plated in a monolayer on a matrix coating such as Matrigel. The cells are then exposed to key molecules necessary to activate or repress transcription factors that direct the cells to EC fate. The ECs are selected using sorting techniques such as fluorescence activated cell sorting (FACS) or magnetic cell sorting. They are then replated onto new dishes for expansion (36).

Orlova et al. (39) developed a 10-day differentiation protocol for ECs and pericytes from human iPSCs. Treating iPSCs with BMP4, activin A, VEGF, and CHIR99021 (CHIR)—a GSK3 inhibitor that activates Wnt signaling—induces mesoderm specification. After 3 days, the media is replaced by vascular specification media supplemented with VEGF and the chemical TGF-β inhibitor SB431542. On day 10, ECs are selected by FACS sorting using CD144 (also known as vascular endothelial cadherin or VE-cadherin) and CD31 as markers.

Patsch et al. observed that treatment with CHIR and BMP4 induces a significant and transient increase in mesoderm markers in human pluripotent stem cells (hPSCs) such as *T, MIXL* and *EOMES* and a decrease in the pluripotent markers *OCT4* and *SOX2* (40). Vascular endothelium is then induced by treating iPSCs induced to mesoderm with VEGF-A and the protein kinase A activator forskolin. ECs are selected using CD144 as a marker. These cells are positive for *SOX17* and EC markers such as CD31, CD34, von Willebrand factor (vWF), and PECAM1. Transcriptomic assays, barrier function, lipid uptake, cytokine response, and tube formation, show their similarity to primary ECs.

ECs can be generated from iPSCs after lateral plate mesoderm development by first inducing the epiblast stage in iPSCs using a combination of FGF2, retinoic acid (RA), CHIR, and BMP4 (41). In this protocol, primitive streak induction is then obtained through prolonged treatment with CHIR, FGF2, and BMP7, and withholding RA and BMP4. Lateral plate mesoderm is then induced by prolonged BMP7 treatment, giving rise to VEGFR2^+^ mesodermal cells. EC specification media, consisting of serum-free Stempro-34 medium supplemented with VEGF and the Wnt/β-catenin signaling inhibitor IWR1, was added to these mesodermal cells. The efficiency was over 90% in CD144^+^ and VEGFR2^+^ iPSC-derived ECs with a 4-day protocol.

### Selective Transcription Factor Expression

Another approach to differentiate iPSCs to ECs involves addition of transcription factors either by gene editing or exogenous expression of genes. Elcheva et al. (42) performed a comprehensive screen and found 27 genes implicated in formation of mesoderm and vascular progenitors. Of these, 14 genes induce morphological changes when virally transduced into hPSCs. Of note, stem cells transduced with either ETV2 or ERG—both potent TFs in determining EC fate—displayed morphology and phenotypes characteristic of ECs such as CD31 and CD34 expression, tube formation in response to VEGF, and acetylated low-density lipoprotein (AcLDL) uptake. Co-transduction with ETV2 and GATA2 resulted in hematopoietic cells *via* an EC state. EC genes were upregulated on the second day after co-transfection, with CD144 expression observable by day 3. The difference between cells transduced with ETV2 alone and those co-transfected with ETV2 and GATA2, comes after day 3 where hemogenic phenotypes become present in co-transduced cells. This implies a role for ETV2 in inducing EC fate directly and sets a precedent for ETV2 transduction as a means for generating stem cell-derived ECs.

Recently, Wang et al. used chemically modified RNA (modRNA) to endogenously express ETV2 during the mesodermal stage of iPSC differentiation, resulting in highly efficient EC production (43). After inducing mesodermal progenitors by the addition of CHIR, modRNA was transfected into mesodermal cells *via* lipofection or electroporation to deliver ETV2. Each step lasted 48 h and resulted in 95% efficiency of CD144 and CD31 positive cells, which was consistent across iPSC clones. They concluded that the robust and transient expression of ETV2 was sufficient to generate ECs independent of endogenous VEGFR2 and ETV2 expression. These ECs function adequately in comparison with ECFCs and ECs generated from a previous protocol (43).

An alternative approach to produce ECs involves reprogramming of primary cells without going through the pluripotent state. This is accomplished by expression of cell-type specific transcription factors together with small molecules and specific growth factors (44). The classic example involves induction of a skeletal muscle phenotype by expression of MYOD in fibroblasts (45). Of a number of transcription factors expressed in ECs, expression of ETV2 was sufficient to induce differentiation of human fibroblasts to ECs (46, 47). Two rounds of ETV2 expression seemed sufficient to produce ECs with gene expression profiles very similar to microvascular and venous ECs (46).

### Function and Characterization of iPSC-Derived Vascular ECs

ECs derived from iPSCs must be properly characterized to ensure the validity of the differentiation protocol and the functionality of the resulting cells. Validation and functional assessment are done in various ways, from immunostaining for EC-specific markers to measuring the barrier function of the derived cells.

Selecting for EC-specific markers is one of the most straightforward preliminary validation methods. CD144 establishes the integrity of EC-EC junctions (48). vWF is made by ECs and secreted to facilitate thrombosis (49). CD31 is a transmembrane glycoprotein which is concentrated at EC junctions (50). CD31, CD144, and VEGFR2 are useful EC markers and can be used in flow cytometry as well to detect and sort ECs (40, 51). Staining for non-EC markers such as smooth muscle actin (SMA) is done as a negative control and to show a pure population of ECs void of SMCs. Uptake of acetylated LDL, a modified form of LDL, is often used to characterize ECs after isolation (52).

ECs exhibit specific behavior in co-culture with vascular smooth muscle cells (VSMCs) that can be used to validate their functionality. During EC differentiation of iPSCs, some cells typically arise that exhibit VSMC phenotypes, as detected by expression of SMA. It is common to use this fraction of cells to induce a VSMC fate using factors different from those used for EC differentiation (39, 40). ECs form an island with VSMCs surrounding them, as well as vascular structures (39). ECs also form vascular tubes when cultured on Matrigel (51). This type of validation illustrates the potential for iPSC-derived ECs to mimic the characteristic behavior of primary ECs and their interactions with their environment.

*In vivo* validation is also done using xenografts and imaging. Transplantation of iPSC derived ECs into zebrafish embryos whose immune systems are not fully developed show incorporation of the cells in the adult zebrafish's vasculature (39).

TNFα signaling in ECs leads to the activation of reactive oxygen species (ROS) and an increase in inflammation (53). TNFα treatment of an EC monolayer can therefore serve as a functional test by observing the inflammatory response of treated ECs. ICAM1 is an inflammatory marker found upregulated in aged blood vessels. ICAM1 upregulation upon TNFα treatment gives an indication that the ECs are responding appropriately.

Validating the function of ECs also includes testing the barrier capabilities of the monolayer using transendothelial electrical resistance (TEER) or observing the permeability of the layer to various molecules. The Lian et al. (51) study used TEER readings and dextran transfer assays to determine EC function under VEGF and o-ME treatment. VEGF treatment caused an increase in TEER and a decrease in dextran permeability. The inverse was true for o-ME treatment to the EC monolayer.

Differences are observed between iPSC-derived ECs and primary ECs. RNA sequencing has shown that while iPSC-ECs show differing expression patterns from stem cells (54), they also do not cluster with primary ECs (55). Key endothelial markers and flow-sensitive genes, such as NOS3, are expressed, but often at lower levels than found in primary ECs (54). Metabolic genes also have significantly different expression in iPS-derived ECs compared to primary ECs (55). Pluripotent markers such as Oct4 and Klf4 (54) have been detected in iPS-derived ECs which signifies some immaturity. Immature mitochondria in iPSC-derived ECs leads to a reduced glycocalyx, a polysaccharide layer involved in protein-receptor interactions, which facilitates and is regulated by shear sensing. Mature mitochondria and the glycocalyx are restored by addition of cyclosporine (55). Despite these differences, iPSC-ECs remain a great resource for research since their functionality recapitulates primary ECs *in vitro*.

## Applications of iPS-Derived Endothelium

**Figure 4:**
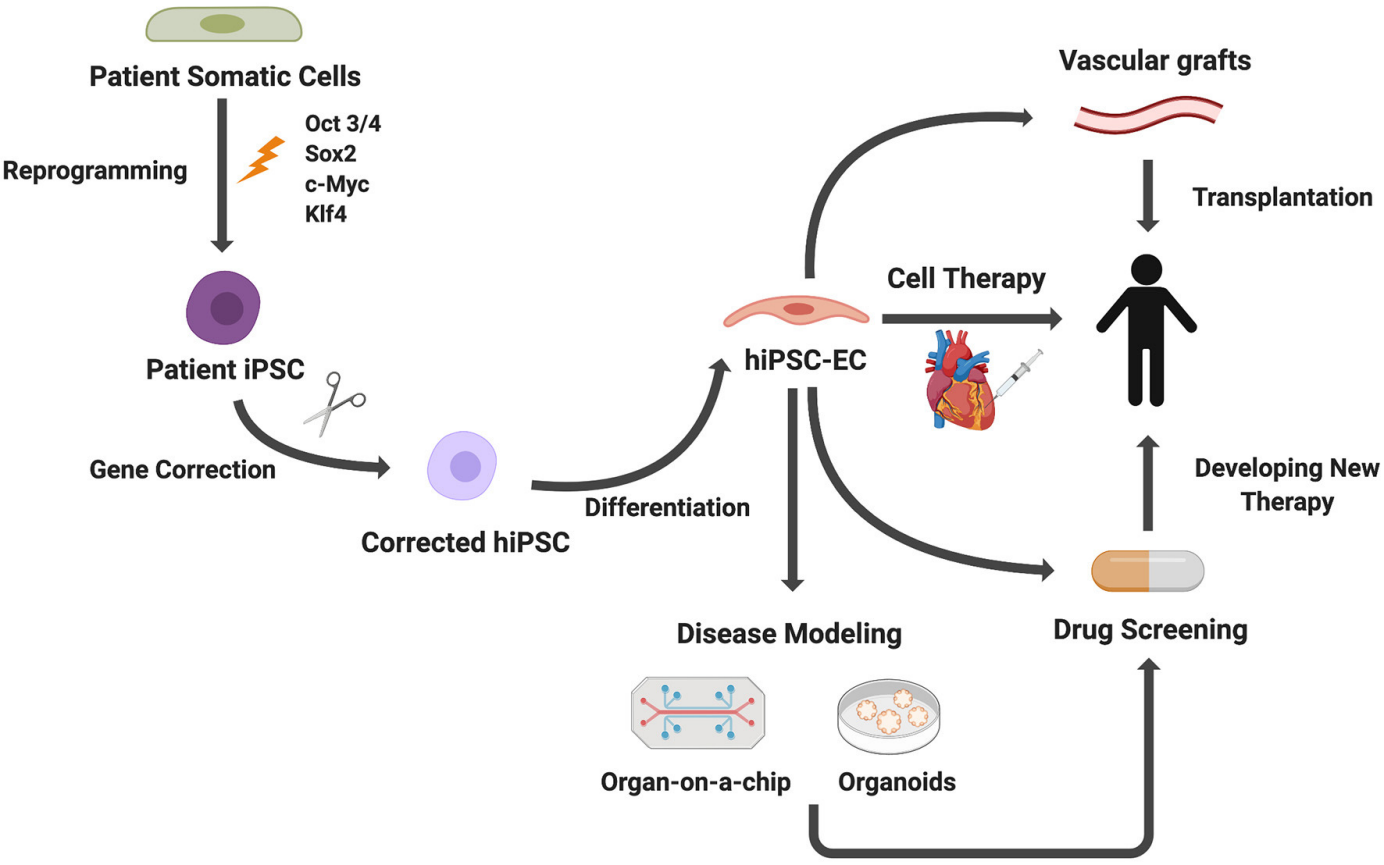
Applications of iPSC-derived ECs. Patient somatic cells are reprogrammed to iPSCs. For genetic diseases, gene editing can be used to correct genetic mutations. ECs differentiated from iPSCs can be used for disease modeling, cell therapies, drug screening, and vascular grafts.

Growing understanding of EC development, leading to the refining of EC differentiation protocols from stem cells, enables generation of ECs for applications in tissue engineering and regenerative medicine (56). Here we present several applications of iPSC-ECs with a particular focus on 3D *in vitro* modeling, including microfluidic devices and organoid models (Figure 4). We then discuss how these *in vitro* models are currently being applied to disease modeling, drug screening, and personalized medicine. Lastly, we will discuss the ongoing efforts to employ iPSC-ECs for therapeutic applications.

### 3D *in vitro* Modeling

3D organ modeling systems represent promising approaches to recapitulate human physiology *in vitro*. Organoids are self-organizing, 3D cellular aggregates that mimic key features of a specific human organ or tissue. Because iPSC-derived organoids demonstrate an intrinsic capacity to self-assemble, they can approximate *in vivo* developmental processes that give rise to the organ of interest (57). Another modeling platform using iPSC-derived cell types, microfluidic organ-on-chips utilize biocompatible materials and microfabrication technology to replicate the microarchitecture and function of specific human organs (58). Unlike 2D cultures of primary or immortalized human cells which have limited biological function, organ-on-a-chip technology better mimics the *in vivo* vascular environment.

#### Microvasculature

One of the key challenges in tissue engineering is that tissue constructs thicker than 100–200 μm must be vascularized in order to ensure a sufficient supply of oxygen and nutrients at core regions of the tissue (59). Toward this end, a number of models are available to form microvascular networks *in vitro*. One approach is to induce the self-assembly of microvascular networks by providing ECs with various chemical, cellular, or biophysical cues to simulate vasculogenesis. ECs and supporting cells (e.g., pericytes, SMCs or fibroblasts) are initially suspended in a scaffold material, which is then cast into a 3D microfluidic chamber and solidified by gelation, polymerization, or crosslinking. Interstitial flow is applied which stimulates ECs to form perfused microvascular networks (60). Alternatively, microvascular models can be formed by seeding ECs onto a preformed supporting structure using 3D printing or channel templates or by embedding cells into a hydrogel and subsequently inducing sprouting *via* flow and chemical factors (i.e., VEGF, hypoxia, nutrient deprivation) (60–62). All of these approaches utilize similar cellular components.

Endothelial colony-forming cells, HUVECs, human dermal microvascular ECs, and iPSC-derived ECs are most often employed in microvasculature models (60). iPSC-ECs form microvascular networks in 3D cultures (61, 62), although limitations still exist in replicating *in vivo* cell and matrix densities and the mechanical properties of native tissues (60). Most models incorporate supporting cells including pericytes, smooth muscle cells, and fibroblasts to improve barrier function, remodel the extracellular matrix (ECM), induce angiogenesis, and stabilize the capillary network (60).

Microvascular systems can be used to model cancer metastasis *in vitro*. Tumor growth requires perfused vascular networks to support increased metabolic demands, and tumor ECs secrete factors which stimulate metastasis (63). As a result, many therapeutic strategies focus on inhibiting angiogenesis. So far, microvascular models have been used to create vascularized microtumors and vascular beds for studying tumor cell extravasation and metastasis (60) and tumor cell interactions with immune cells (64).

#### Blood–Brain Barrier

The blood–brain barrier (BBB) consists of brain microvascular ECs (BMVECs) that interact with pericytes and astrocytes to tightly regulate the movement of ions, molecules, and cells between the blood and the central nervous system (CNS). In contrast to peripheral ECs, BMVECs are characterized by the absence of fenestrae, a reduced level of pinocytic vesicles, and a junctional complex formed by tight junctions (TJs) and adherens junctions (AJs) (65). The TJs between BMVECs lead to high endothelial transcellular electrical resistance and low paracellular permeability (66). Because of this impermeable paracellular barrier, the movement of hydrophilic and lipophilic molecules is tightly controlled through regulated cellular transport systems (67). A combination of physical barrier properties (TJs), efflux transporters, and highly specific nutrient transporters allow BMVECs to regulate CNS homeostasis. Alterations of the BBB properties play a major role in the progression of various neurological diseases. As such, there is a need for an *in vitro* BBB model to elucidate the BBB's role in the pathogenesis of neurological diseases in addition to drug screening to assess the efficacy of targeted drug therapies.

Various types of BBB models mimic the structure and physiology of the BBB *in vivo* (68). In 2D-based microfluidic models, ECs and glial/neuronal cells are cultured on a porous membrane or hydrogel surface sandwiched between two microchannels (“blood” side with media perfusion and “brain” side with neural cells in contact with BMVEC). Within the vascular channel, ECs are continuously exposed to fluid flow *via* a peristaltic pump. Fluidic shear stress promotes the formation of a tight barrier, increases cell proliferation, and increases the levels of TJ and AJ proteins in ECs (69). For these reasons, flow-induced shear stress is an essential component for recapitulating *in vivo* BBB characteristics (68). Other groups have modeled the BBB using a 3D hydrogel-based microfluidic chip. By embedding perivascular and neural/glial cells in the hydrogel, this approach better mimics the 3D microenvironment in which these cells reside (68).

Given that the source of human BMVECs is limited, and primary BMVECs possess a low yield and are prone to de-differentiate in culture (70), iPSC-ECs pose an advantage over primary cell types for BBB models. For one, iPSC-ECs are capable of organizing into complex and perfusable vascular networks (71) and exhibit many properties of the human BBB, including ordered tight junctions, expression of nutrient transporters, and polarized efflux transporter activity (72, 73). Additionally, the permeability barrier of these human iPSC-BMVECs exhibit TEER values at physiological levels (72, 74). Differentiation protocols for iPSC-derived BMVECs include a 2-day BMVEC specification stage following endothelial induction. Cells are cultured in human endothelial serum-free medium (hESFM) containing 1% platelet-poor plasma-derived serum, FGF2, and RA (75, 76). The addition of RA during BMVEC specification is a relatively recent advancement in iPSC-BMVEC differentiation and substantially increases the differentiation efficiency and passive barrier properties of iPSC-BMVECs (77). A limitation is that the iPSC-derived BMVECs only exhibit appropriate properties for a limited time.

Several groups have designed BBB models utilizing iPSC-BMVECs, culturing with other various cell types to recapitulate the BBB microvasculature. Campisi et al. developed a 3D self-organized microfluidic model incorporating self-assembled microvascular networks from human iPSC-BMVECs in contact with human primary brain pericytes (PCs) and human primary astrocytes (ACs) within a fibrin gel (71). This BBB model is formed *via* vasculogenesis whereby perfusable microvascular networks interact through paracrine, juxtracrine, and mechanical signaling, thereby drawing upon the nature of BMVECs to interact with other neural cell types to recapitulate brain vascular morphogenesis. Co-culturing the three cell types resulted in more stable and shorter vessel branches, more circular cross-sections, and smaller vessel diameters compared to culturing with PCs or just iPSC-BMVECs. Consequently, they suggest that PCs not only influence vascularization but also induce the differentiation of iPSC-ECs into brain microvascular ECs, while ACs contribute to BBB formation and integrity. While this model demonstrated low permeability compared to other models (78, 79), they were limited to assessing permeability using a fluorescently labeled molecular permeability assay given that fibrin gel creates challenges with incorporating electrodes for high sensitivity TEER measurements.

iPSC-BMVECs co-cultured with iPSC-derived neural cells showed similar dextran-FITC permeability as iPSC-BMVECs co-cultured with primary astrocytes and pericytes, suggesting that iPSC-derived neural cultures without pericytes are sufficient to drive functional maturation of iPSC-BMVECs (75). Moreover, the BBB chip exhibited physiologically relevant TEER measurements as high as 1,500 Ω cm^2^. However, the proposed model could only maintain these high TEER levels for around 2 days. To resolve this limitation, Park et al. developed a 3D microfluidic model consisting of iPSC-BMVECs differentiated under hypoxic conditions, along with primary pericytes, and astrocytes (72). Mimicking the hypoxic microenvironment of the developing brain during differentiation of human iPSCs into BMVECs resulted in more highly differentiated iPSC-BMVECs with a more stable phenotype. The resulting BBB chip exhibited physiologically relevant TEER values for 1 week *in vitro* compared to chips containing iPSC-BMVECs established under normoxic conditions, in which TEER values only remained physiologically relevant for 2–3 days. Even more notably, the BBB chip was able to mimic transporter-mediated drug efflux with appropriate substrate specificity.

While iPSC-BMVECs mimic many phenotypic and functional features of *in vivo* BMVECs, several differences between iPSC-derived and *in vivo* BMVECs remain (77). The iPSC-BMVECs express several epithelial markers and may not possess a purely endothelial identity (75), emphasizing the need for the optimization of iPSC-BMVEC differentiation protocols. Moreover, the expression of some transporters often falls below physiological levels (75), indicating a need for further iPSC-BMVEC maturation or altered culturing conditions (77). Moreover, while organ-on-chip technology has enabled truer representations of the *in vivo* BBB, researchers are still limited by their inability to completely recapitulate the brain microenvironment, including ECM composition as well as the small diameters, shear stresses, and complex vascular networks observed in brain capillaries (68, 77). However, with further innovations in iPSC technology, biomaterials, and microfluidics, 3D BBB models should prove invaluable in the study of neurodegenerative diseases and drug discovery.

#### Liver

ECs provide the potential to support liver organogenesis and improve tissue functionality (56). iPSC-derived hepatic endoderm cultured in the presence of human umbilical vein endothelial cells (HUVECs) and mesenchymal stem cells form a 3D vascularized liver organoid that supports further hepatic differentiation (80). When evaluating the functional maturation of iPSC-derived liver bud (LB) transplants in mice compared with that of human primary adult hepatocytes (AH), the iPSC-LBs maintain higher levels of serum albumin production over a 45-day period. Moreover, transplanted human iPSC-derived mature hepatocyte-like cells produce significantly less albumin than either iPSC-LBs or human AH, suggesting the importance of the formation of 3D vascularized tissue for successful maturation and engraftment. The maturation of these liver organoids is regulated by the paracrine signals produced by mesenchymal stem cells and HUVECs, which promote hepatic differentiation in iPSC-derived human endoderm (81). Single-cell RNA sequencing to reconstruct hepatocyte lineage progression during organogenesis indicates that hepatic cell maturation is improved in the presence of HUVECs (82).

The lack of complex *in vitro* liver models including mature human hepatocytes remains a major hurdle for the pharmaceutical industry to provide low-cost drug and liver toxicity testing (83).The development of these models *in vitro* often necessitates the incorporation of hepatic stellate cells (HSCs) and liver sinusoidal endothelial cells (LSECs). As mentioned previously, LSECs differ from classical ECs in that they lack an organized basement membrane and have fenestrae organized in sieve plates (84). Additionally, compared to vascular ECs, they exhibit reduced levels of expression of CD34, CD31 (*PECAM1*), and vWF and elevated expression of LYVE1, CD14, CD32B (*FCGR2B*), CD36, CD54, STAB1, STAB2, CLEC1B, and factor VIII (FVIII) (85). Maintenance of these cells *in vitro* remains challenging as primary LSECs tend to rapidly lose some of their specific properties, including the expression of LSEC markers and the presence of fenestrae (84). In this regard, iPSCs are now being considered as an alternative source of these cells. However, while iPSCs have been differentiated toward an LSEC phenotype (83, 85), information about the LSEC signature and its transcriptional determinants remains incomplete (84). Looking to further extend the characterization of iPSC-derived LSECs, Danoy et al. performed whole transcriptome analysis with nanoCAGE and compiled the regulatory network involving the relevant transcriptional factors and their target genes and related signaling pathways (83). Likewise, de Haan et al. established a 30-gene LSEC signature, including seven genes encoding TFs, by comparing isolated LSECs with ECs from the brain and heart. They also tested the individual and combined effect of LSEC TFs on LSEC gene expression, morphology, and function (84). Thus, the development of more complex *in vitro* hepatic multi-cellular tissues is largely dependent on uncovering the developmental origins of LSECs and the pathways that control their specification and maturation (85). In an effort to address some of the challenges of current liver models, including variable performance and high manufacturing costs, Lucendo-Villarin et al. (86) developed an automated and economical platform to produce 3D liver spheroids by combining hepatic progenitors with ECs and stellate cells. The resulting system, which can be employed for disease modeling and small molecule screening, represents an advancement towards what will one day hopefully become large-scale manufacturing of functional 3D human liver tissue for *in vitro* and clinical applications.

#### Kidney

iPSC-derived kidney organoids offer a tool to study human kidney development and disease. However, significant barriers remain for these organoids, one of which is lack of vascularization. While blood flow stimulates kidney vascularization (87), most glomeruli within kidney organoids are avascular *in vitro* (88), highlighting the limitations of current *in vivo* culture methods (89). As one exception, Homan et al. demonstrated a method to culture kidney organoids under flow on a microfluidic device, which promoted the formation of vascular networks with perfusable lumens inside the organoids and expanded the endogenous pool of endothelial colony-forming cells (90).

The formation of vascular networks in kidney organoids is enhanced following transplantation under the kidney capsule of immunodeficient mice (89, 91, 92). Following transplantation, the mouse vasculature connects to pre-existing vasculature in the human kidney organoids to enable functional glomerular perfusion (92). At this point, the necessity of incorporating ECs in kidney organoids before transplantation remains unclear. A large part of this uncertainty stems from a lack of understanding of the contribution of donor and host-derived ECs within transplanted kidney organoids. Most ECs in the glomeruli post-implantation are derived from the host animals and there is partial integration of iPSC-derived ECs upon transplantation (93, 94). As a result, further studies are needed to elucidate whether ECs need to be derived from human iPSCs, and whether kidney-specific ECs or non-tissue specific ECs suffice (88).

However, in models of the kidney proximal tubule (PT), a key component of the nephron that is particularly susceptible to nephrotoxicity (95), microfluidic and tissue engineering platforms have advantages over kidney organoids. Organoids are limited in size without adequate vascularization (57), and unlike microfluidic models, they lack defined inlet and outlets, making it difficult to collect and analyze their perfusate (96). Though technically challenging, kidney segment-specific platforms bypass these limitations and can serve as useful tools for disease modeling and drug screening. To date, both microfluidic (95, 97) and bioprinted PT models (96, 98) have been developed. These models aim to recapitulate the tubular-vascular exchange by seeding PT and primary ECs on either side of a synthetic membrane. No published work, to our knowledge, has yet described a kidney-on-a-chip model with iPSC-ECs.

#### Blood Vessel Organoids

Apart from microphysiological systems, blood vessel organoids provide another approach to study vascular diseases. Though similar in their aim to recapitulate the vasculature *in vitro*, vascular microphysiological systems and blood vessel organoids are conceptually different. A vascular microphysiological system consists of a microfabricated device with microfluidic channels that enables close contact between different cell types and physical and biochemical stimuli, such as flow and pressure. Vascular organoids, on the other hand, consist of self-organizing 3D vascular networks which better reflect the inherent complexity of the cellular environment (99). Human blood vessel organoids can be generated through the directed differentiation of iPSCs to self-assembled human blood vessel organoids, bypassing the need for cell sorting during the differentiation process (100). These miniaturized blood vessel organoids closely resemble human capillaries with a lumen, CD31^+^ endothelial lining, PDGFR^+^ pericyte coverage, and basement membrane. Once generated, the blood vessel organoids were used to model diabetic vasculopathy. Following transplantation into immunocompromised mice, the organoids gain access to the mouse vasculature to form a stable, fully human vasculature consisting of arteries, capillaries, and veins. In regard to future applications, blood vessel organoids could prove particularly valuable in conjunction with vascularized kidney organoids to study interorgan communication in diabetic conditions (101). Additionally, they can be used to assess vascular toxicity as well as model rare, genetic vascular diseases (102). In contrast to microfluidic models of microvascular networks, blood vessel organoids offer the unique advantage of recapitulating key steps of *in vivo* blood vessel development. As a result, several key components of successful blood vessel growth can be examined, including endothelial differentiation and sprouting into vascular networks, pericyte differentiation and recruitment, and the formation of a basement membrane (102).

### Disease Modeling

Human iPSCs are an attractive source for disease modeling as they can easily be expanded and cultured prior to EC differentiation and can often be obtained through the programming of somatic cells from human subjects. Notably, iPSC-ECs can also be used to create patient-specific disease models, enabling a platform for precision medicine. Disease models can be generated using patient-specific cells that account for differences due to genetic diversity, sex, ethnicity, and age (103). Such an approach could allow for the development of optimized, personalized drug regimens for patients as well as prognostic disease models. iPSC disease models are particularly useful for rare genetic disorders where the donor pool is limited, or for patient populations typically unfit for standard clinical trial designs. With the use of gene editing techniques such as CRISPR-Cas9, iPSCs can be used to study how specific gene mutations affect tissue functionality and can provide new insights into the progression of diseases and mechanisms of drug action (103).

The pathogenesis of vascular diseases is typically associated with the alteration of vascular ECs and SMCs, making cells of the vessel wall attractive targets for pathogenesis and drug response studies. Disease-specific iPSC-ECs have been generated to model several vascular disorders, including pulmonary arterial hypertension, Moyamoya disease, fibrodysplasia ossificans progressiva, Huntington's disease, Kawasaki disease, type I diabetes mellitus, atrial or ventricular septal defects, pulmonary valve stenosis, cardiomyopathy, calcified aortic valve disease, and hemophilia A as previously summarized (36). Most of these diseases have a genetic basis and are therefore less challenging to model *in vitro* than diseases that are more heterogeneous in cause. In these studies, disease models were used to study the effects of specific mutations on EC phenotype in order to elucidate the role of EC dysfunction in overall disease pathology. For example, iPSC-ECs were generated to study the BMPR2 (bone morphogenetic protein receptor type II) mutation that causes pulmonary arterial hypertension (PAH) and PAH-specific iPSC-ECs exhibited decreased tube formation, LDL uptake (104), adhesion, migration, and survival (105). iPSC-ECs from patients with Moyamoya disease carrying the RNF213 mutation exhibited reduced angiogenic activity (106). Such studies demonstrate how iPSC-ECs can be used to explore the underlying molecular mechanisms of CVD. In addition to functional assays, disease-specific iPSC-ECs have been examined by RNA-sequencing. Transcriptome analysis of BMVECs in BBB dysfunction in Huntington's disease identified a set of BMVEC genes that could impact normal and diseased BBB function (107). These studies highlight how iPSC-ECs can serve as useful surrogates to uncover novel features of disease pathologies, thereby paving the way for the development of new therapies.

Recently, Atchison et al. developed a tissue-engineered blood vessel (TEBV) platform for disease modeling of Hutchinson-Gilford Progeria Syndrome (HGPS) (108), a rare genetic condition which produces a truncated form of lamin A called progerin. Progerin accumulation, which occurs primarily in vascular SMCs but also adventitial fibroblasts and ECs, results in accelerated aging and premature atherosclerosis. To investigate the effects of progerin on ECs, Atchison et al. (108) developed TEBVs using SMCs and ECs derived from the same HGPS iPSC line. They observed that in 2D culture, HGPS ECs exhibited reduced responsiveness to flow as well as altered levels of NOS3, which regulates nitric oxide production. In 3D conditions, they found that regardless of whether healthy or diseased SMCs were incorporated into the TEBV constructs, there was still a decrease in vasodilation in TEBVS that incorporated HGPS ECs. Finally, they found that HGPS ECs produced the adhesion proteins VCAM1 and E-selectin regardless of the SMC donor. The results of this study indicate a role for the endothelium in HGPS while providing a unique model system to investigate HGPS aging pathologies.

A patient-specific iPSC model was recently developed by Kelleher et al. for CADASIL (cerebral autosomal dominant arteriopathy with subcortical infarcts and leukoencephalopathy), a rare genetic small vessel disease that causes stroke and vascular dementia (109). The disease is characterized by SMC degeneration and accumulation of NOTCH3 extracellular domain proteins. Moreover, abnormal ECs and reduced vasodilation in the arteries of CADASIL patients indicate EC involvement in CADASIL. Kelleher et al. hypothesized that the communication between ECs and mural cells (MCs; pericytes and SMCs) through the Notch signaling pathway was disrupted by the NOTCH3 mutation present in CADASIL. iPSCs derived from CADASIL patients were differentiated into ECs and MCs to generate a patient-specific model. Yet CADASIL iPSC-MCs failed to stabilize angiogenic capillary tubule structures and support iPSC-EC survival. Additionally, these diseased iPSC-MCs had a reduced expression of PDGFRb (platelet-derived growth factor receptor b), a key pericyte marker, and reduced expression and secretion of VEGF. This disease model provides a potential tool for investigating the mechanisms underpinning CADASIL and vascular dementia as well as a potential drug screening platform.

In patients with diabetes, endothelial dysfunction appears to be a consistent outcome, yet the relationship between diabetes and endothelial function is still unclear (110). This is particularly true with respect to the pathological activation of calpain, an intracellular cysteine protease, whose over-activation causes EC dysfunction and inflammatory responses in patients with diabetes mellitus. Ong et al. developed a model of diabetic endotheliopathy utilizing iPSC-ECs in order to understand how the changes to autophagy and mitochondrial dynamics caused by hyperglycemia leads to EC dysfunction (110). iPSC-ECs exposed to hyperglycemia exhibited reduced autophagy, increased mitochondria fragmentation, and increased calpain activity. However, calpain inhibition reversed these effects and resulted in improved vascular integrity.

A recent study investigated how the blood–brain barrier (BBB) was altered in familial Alzheimer disease (AD) (111). Oikari et al. derived brain endothelial cells (iBECs) from iPSCs of patients carrying the AD PSEN1 mutation in order to study the differences between AD and healthy ECs. They found that AD-iBECs exhibit altered tight and adherens junction protein expression as well as efflux properties, supporting the idea of an impaired BBB in AD.

### Drug Screening

With the imminent arrival of personalized medicine, iPSCs offer an attractive platform for high-throughput drug and toxicity screening. Drug development is often an inefficient, resource-intensive process with the cost required to complete the entire drug development process estimated to be over $2.5 billion (112). Neurotoxicity, hepatotoxicity, and cardiovascular toxicity all represent major causes of the failure of drugs in clinical trials and post market withdrawal (57). Currently, most drug studies rely on simple human cell cultures or animal models. However, performing drug screening on primary patient-derived cells has proven to be challenging due to their limited availability and batch-to-batch variability (113). Moreover, the limitations of animal models due to species-specific differences in physiology, metabolism, and genetics can result in inaccurate (false-positive or false-negative) readouts in preclinical drug safety or efficacy studies and ultimately lead to drug failure during clinical trials (57, 103). iPSC technology coupled with organ-on-a-chip (OOC) platforms, could present a more accurate, efficient, and sophisticated method of drug development. iPSC-OOCs can be used in preclinical drug screening to confirm both the absence of toxicity and the efficacy of treating targeted pathways. Consequently, iPSC-OOCs can inform decisions about which drug's development should be advanced and which should be halted (103).

iPSC-ECs specifically provide the ability to study the endothelium's response to drug. In 2D, iPSC-ECs have been studied in the context of statins, a class of HMG-CoA reductase inhibitors, which evoke KLF2 expression and a non-thrombogenic and anti-inflammatory phenotype (114). iPSC-ECs have been used to evaluate the toxicity of chemotherapeutic compounds such as tyrosine kinase inhibitors, which elicit cardiac and endothelial toxicity (57, 115). Additionally, Vazao et al. (116) used iPSC-ECs to screen for drugs that affect vascular embryonic development.

More recently, a 3D tumor-on-a-chip model was developed to assess the cardiotoxicity of the anticancer drugs doxorubicin and oxaliplatin (117). The model, which consisted of tumor cell spheroids, iPS-derived cardiomyocytes (iPS-CMs), and iPSC-ECs evaluated the effect of these drugs on the spontaneous beat rate and conduction velocity of iPS-derived cardiac tissue. Consistent with *in vivo* observations, doxorubicin reduced the spontaneous beat rate and maximum capture rate at or near the drug's inhibition concentration (IC_50_), whereas oxaliplatin-induced toxicity occurred at concentrations significantly higher than the IC_50_. This study also illustrates how co-culture with multiple cell types through OOCs can prove valuable for evaluating drugs that can produce both cardiac and endothelial toxicity.

Of late, drug screening of engineered tissues is valuable in tackling the COVID-19 pandemic. A recent study showed that human blood vessel organoids and kidney organoids can be readily infected by SARS-CoV-2, but early-stage infection can be inhibited by clinical-grade human recombinant soluble ACE2 (hrsACE2) (118). This data suggests that soluble recombinant human ACE2, which has already been tested in phase 1 and phase 2 clinical trials, may prove to be a viable option in blocking the effects of the SARS-CoV-2 virus.

### Therapeutic Applications

iPSC-ECs were initially produced as a means of investigating their potential as a cell-based therapy for the treatment of vascular diseases. Many cases of CVD, including coronary and peripheral heart disease, diabetes mellitus, and stroke, are characterized by ischemia, a reduction of oxygenated blood supplied to tissues, leading to necrosis (59, 119). Cell-based therapies aim to rebuild vasculature by promoting angiogenesis and vasculogenesis in ischemic tissues. iPSC-ECs, which form functional blood vessels after transplantation in animal models (120), constitute a potential source of autologous cells for such therapies. For example, iPSC-ECs have been administered intramuscularly in murine hindlimb ischemia models and have been found to enhance wound angiogenesis and limb perfusion (121). However, direct injection of iPSC-ECs typically has a low rate of *in vivo* cell survival and poor cell function (122). One strategy used to overcome these limitations is to suspend ECs in hydrogels or synthetic biomaterial scaffolds before injection (122, 123).

In addition to providing perfusion in ischemic tissues, iPSC-ECs can also be used to generate microvascular networks within tissue grafts. Recent approaches can be divided into two basic strategies as detailed extensively by Song et al. (124): (1) angiogenic sprouting or vasculogenic self-assembly and (2) fabrication of pre-designed structures that are then endothelialized (124). To date, both approaches have been used to vascularize engineered tissues, including liver (82, 125), skin (126) and skeletal muscle (127).

There has also been interest in utilizing iPSC-ECs in the development of larger tissue-engineered blood vessels for various cardiovascular applications, such as bypass grafting, as well as arteriovenous grafts for hemodialysis access. Commercial synthetic grafts suffer from low patency and high failure rates, prompting interest in developing small-diameter vascular grafts that resist thrombosis, inflammation, and aneurysm formation (128). While one study has utilized iPSC-ECs and iPSC-SMCs to generate bilayered vascular grafts (129), whether or not iPSCs will be adopted in larger-scale TEBVs for therapeutic purposes remains to be seen. One barrier to employing iPSC-based tissue-engineered vascular grafts is the extensive time needed for iPSC expansion and differentiation, which greatly limits clinical feasibility. One alternative solution has been to seed biodegradable scaffolds with mesenchymal stem cells, which, unlike iPSCs, can be harvested and cultured on a clinically relevant time scale. Krawiec et al. (130, 131) developed a method to rapidly isolate a fraction of stromal cells from healthy and diabetic donors with stem cell-like behavior to populate vascular grafts. These adipose stem cell-derived grafts were able to remodel *in vivo* and stained positive for vWF and α-SMA demonstrating the presence of an endothelium and smooth muscle cells. While these results are promising, future studies, including those that investigate the long-term outcomes of this technology in more clinically relevant models, are necessary before stem-cell based tissue-engineered vascular grafts become a clinical reality.

## Conclusion and Future Directions

ECs play a critical role in maintaining vascular homeostasis and controlling the blood supply. These cells regulate vascular tone, structure, and inflammation, and their dysfunction plays a key role in instigating atherosclerosis. While primary ECs have limited plasticity and proliferative capacity, iPSCs offer a potentially unlimited source to generate ECs for a wide variety of applications including microphysiological systems, organoids, disease modeling, and cell therapies. Several groups have developed multiple methods to differentiate ECs from iPSCs by mimicking the developmental process of lateral mesoderm to endothelial specification. This can be accomplished by generating EBs, applying precisely timed small molecule treatments to cells cultured in a monolayer, or modulating genetic transcriptional activation in iPSCs. iPSC-ECs display various characteristics consistent with primary ECs including EC marker expression, lipid uptake, barrier function, and tube formation. These cells have already been used in high throughput drug and toxicity screening, modeling human vascular diseases, vascularizing organoids, and creating tissue-specific *in vitro* models including BBB, liver, kidney, and blood vessels.

Several important challenges remain to be addressed in future studies. The ability of iPSC-ECs to improve organoid vascularization and whether tissue specificity is needed for this application remains to be discovered. Further, while they share functional similarities, iPSC-ECs and primary ECs still differ significantly in their gene expression. For example, iPSC-BMVECs share phenotypic and functional features with *in vivo* BMVECs making them valuable tools in creating BBB models, but they also express epithelial markers and reduced expression of some BMVEC transporters. Much progress is needed in improving the maturation of iPSC-ECs and in developing tissue-specific iPSC-ECs for 3D *in vitro* modeling. Future studies may elucidate what key regulators have not been identified that can improve iPSC-EC phenotype and determine the level of maturity necessary for iPSC-EC applications.

## Author Contributions

CK: wrote first draft, prepared Figure 1, and edited manuscript. GT: conceived of idea, edited manuscript, met with co-authors to review progress, and revised the manuscript. NA: wrote sections on differentiation of iPS cells to ECs, prepared Figure 2, and edited the manuscript. EB: wrote the section Applications of iPS-Derived Endothelium, prepared Figures 3, 4, and edited the manuscript. All authors contributed to the article and approved the submitted version.

## Conflict of Interest

The authors declare that the research was conducted in the absence of any commercial or financial relationships that could be construed as a potential conflict of interest.

## References

[1] Yuan SY Rigor RR. Regulation of Endothelial Barrier Function. San Rafael, CA: Morgan and Claypool Life Sciences (2011). 21634066

[2] Augustin HG Koh GY. Organotypic vasculature: from descriptive heterogeneity to functional pathophysiology. Science. (2017) 357: eaal2379. 10.1126/science.aal2379 28775214

[3] Marcelo KL Goldie LC Hirschi KK. Regulation of endothelial cell differentiation and specification. Circ Res. (2013) 112:1272–87. 10.1161/CIRCRESAHA.113.300506 23620236 PMC3768127

[4] Hosoya T Maruyama A Kang MI Kawatani Y Shibata T Uchida K. Differential responses of the Nrf2-Keap1 system to laminar and oscillatory shear stresses in endothelial cells. J Biol Chem. (2005) 280:27244–50. 10.1074/jbc.M502551200 15917255

[5] Gimbrone MA Jr Garcia-Cardena G. Endothelial cell dysfunction and the pathobiology of atherosclerosis. Circ Res. (2016) 118:620–36. 10.1161/CIRCRESAHA.115.306301 26892962 PMC4762052

[6] Dogné S Flamion B Caron N. Endothelial glycocalyx as a shield against diabetic vascular complications. Arterioscler Thromb Vasc Biol. (2018) 38:1427–39. 10.1161/ATVBAHA.118.310839 29880486 PMC6039403

[7] Montagne A Zhao Z Zlokovic BV. Alzheimer's disease: a matter of blood–brain barrier dysfunction? J Exp Med. (2017) 214:3151–69. 10.1084/jem.20171406 29061693 PMC5679168

[8] Souilhol C Serbanovic-Canic J Fragiadaki M Chico TJ Ridger V Roddie H. Endothelial responses to shear stress in atherosclerosis: a novel role for developmental genes. Nat Rev Cardiol. (2020) 17:52–63. 10.1038/s41569-019-0239-5 31366922

[9] Zhou J Li YS Chien S. Shear stress-initiated signaling and its regulation of endothelial function. Arterioscler Thromb Vasc Biol. (2014) 34:2191–8. 10.1161/ATVBAHA.114.303422 24876354 PMC4169328

[10] Sekiya S Shimizu T. Introduction of vasculature in engineered three-dimensional tissue. Inflamm Regen. (2017) 37:25. 10.1186/s41232-017-0055-4 29259724 PMC5725988

[11] Vyas C Pereira R Huang B Liu F Wang W Bartolo P. Engineering the vasculature with additive manufacturing. Curr Opin Biomed Eng. (2017) 2:1–13. 10.1016/j.cobme.2017.05.008

[12] Adams RH Alitalo K. Molecular regulation of angiogenesis and lymphangiogenesis. Nat Rev Mol Cell Biol. (2007) 8:464–78. 10.1038/nrm2183 17522591

[13] Corada M Morini MF Dejana E. Signaling pathways in the specification of arteries and veins. Arterioscler Thromb Vasc Biol. (2014) 34:2372–7. 10.1161/ATVBAHA.114.303218 25169934

[14] Corada M Nyqvist D Orsenigo F Caprini A Giampietro C Taketo MM. The Wnt/beta-catenin pathway modulates vascular remodeling and specification by upregulating Dll4/Notch signaling. Dev Cell. (2010) 18:938–49. 10.1016/j.devcel.2010.05.006 20627076 PMC8127076

[15] Reis M Liebner S. Wnt signaling in the vasculature. Exp Cell Res. (2013) 319:1317–23. 10.1016/j.yexcr.2012.12.023 23291327

[16] Lawson ND Scheer N Pham VN Kim CH Chitnis AB Campos-Ortega JA. Notch signaling is required for arterial-venous differentiation during embryonic vascular development. Development. (2001) 128:3675–83 11585794 10.1242/dev.128.19.3675

[17] Lee D Park C Lee H Lugus JJ Kim SH Arentson E. ER71 acts downstream of BMP, Notch, and Wnt signaling in blood and vessel progenitor specification. Cell Stem Cell. (2008) 2:497–507. 10.1016/j.stem.2008.03.008 18462699 PMC2683414

[18] Montesano R Vassalli JD Baird A Guillemin R Orci L. Basic fibroblast growth factor induces angiogenesis *in vitro*. Proc Natl Acad Sci USA. (1986) 83:7297–301. 10.1073/pnas.83.19.7297 2429303 PMC386703

[19] Ding VM Ling L Natarajan S Yap MG Cool SM Choo AB. FGF-2 modulates Wnt signaling in undifferentiated hESC and iPS cells through activated PI3-K/GSK3beta signaling. J Cell Physiol. (2010) 225:417–28. 10.1002/jcp.22214 20506199

[20] Dvorak P Hampl A. Basic fibroblast growth factor and its receptors in human embryonic stem cells. Folia Histochem Cytobiol. (2005) 43:203–8. 16382885

[21] Kelly MA Hirschi KK. Signaling hierarchy regulating human endothelial cell development. Arterioscler Thromb Vasc Biol. (2009) 29:718–24. 10.1161/ATVBAHA.109.184200 19213939 PMC2729243

[22] Astorga J Carlsson P. Hedgehog induction of murine vasculogenesis is mediated by Foxf1 and Bmp4. Development. (2007) 134:3753–61. 10.1242/dev.004432 17881493

[23] Goldie LC Nix MK Hirschi KK. Emrbyonic vasculogenesis and hematopoietic specification. In: Ruhrberg C, editor. Development VEGF. New York, NY: Springer-Verlag (2008). p. 40–51. 10.4161/org.4.4.7416 PMC2634331 19337406

[24] Roy H Bhardwaj S Yla-Herttuala S. Biology of vascular endothelial growth factors. FEBS Lett. (2006) 580:2879–87. 10.1016/j.febslet.2006.03.087 16631753

[25] Papapetropoulos A Garcia-Cardena G Madri JA Sessa WC. Nitric oxide production contributes to the angiogenic properties of vascular endothelial growth factor in human endothelial cells. J Clin Invest. (1997) 100:3131–9. 10.1172/JCI119868 9399960 PMC508526

[26] Park C Afrikanova I Chung YS Zhang WJ Arentson E Fong Gh G. A hierarchical order of factors in the generation of FLK1- and SCL-expressing hematopoietic and endothelial progenitors from embryonic stem cells. Development. (2004) 131:2749–62. 10.1242/dev.01130 15148304

[27] Rasmussen TL Shi X Wallis A Kweon J Zirbes KM Koyano-Nakagawa N. VEGF/Flk1 signaling cascade transactivates Etv2 gene expression. PLoS ONE. (2012) 7: e50103. 10.1371/journal.pone.0050103 23185546 PMC3501484

[28] Kataoka H Hayashi M Nakagawa R Tanaka Y Izumi N Nishikawa S. Etv2/ER71 induces vascular mesoderm from Flk1+PDGFRalpha+ primitive mesoderm. Blood. (2011) 118:6975–86. 10.1182/blood-2011-05-352658 21911838

[29] Shah AV Birdsey GM Randi AM. Regulation of endothelial homeostasis, vascular development and angiogenesis by the transcription factor ERG. Vascul Pharmacol. (2016) 86:3–13. 10.1016/j.vph.2016.05.003 27208692 PMC5404112

[30] Nikolova-Krstevski V Yuan L Le Bras A Vijayaraj P Kondo M Gebauer I. ERG is required for the differentiation of embryonic stem cells along the endothelial lineage. BMC Dev Biol. (2009) 9:72. 10.1186/1471-213X-9-72 20030844 PMC2803788

[31] McLaughlin F Ludbrook VJ Cox J von Carlowitz I Brown S Randi AM. Combined genomic and antisense analysis reveals that the transcription factor Erg is implicated in endothelial cell differentiation. Blood. (2001) 98:3332–9. 10.1182/blood.v98.12.3332 11719371

[32] Wythe JD Dang LT Devine WP Boudreau E Artap ST He D. ETS factors regulate Vegf-dependent arterial specification. Dev Cell. (2013) 26:45–58. 10.1016/j.devcel.2013.06.007 23830865 PMC3754838

[33] Niklason L Dai G. Arterial venous differentiation for vascular bioengineering. Annu Rev Biomed Eng. (2018) 20:431–447. 10.1146/annurev-bioeng-062117-121231 29641908 PMC9235071

[34] Wang HU Chen ZF Anderson DJ. Molecular distinction and angiogenic interaction between embryonic arteries and veins revealed by ephrin-B2 and its receptor Eph-B4. Cell. (1998) 93:741–53. 10.1016/s0092-8674(00)81436-1 9630219

[35] Udan RS Vadakkan TJ Dickinson ME. Dynamic responses of endothelial cells to changes in blood flow during vascular remodeling of the mouse yolk sac. Development. (2013) 140:4041–50. 10.1242/dev.096255 24004946 PMC3775417

[36] Lin Y Gil CH Yoder MC. Differentiation, evaluation, and application of human induced pluripotent stem cell-derived endothelial cells. Arterioscler Thromb Vasc Biol. (2017) 37:2014–25. 10.1161/ATVBAHA.117.309962 29025705

[37] Xu M He J Zhang C Xu J Wang Y. Strategies for derivation of endothelial lineages from human stem cells. Stem Cell Res Ther. (2019) 10:200. 10.1186/s13287-019-1274-1 31286997 PMC6615090

[38] Lin B Kim J Li Y Pan H Carvajal-Vergara X Salama G. High-purity enrichment of functional cardiovascular cells from human iPS cells. Cardiovasc Res. (2012) 95:327–35. 10.1093/cvr/cvs185 22673369 PMC4415083

[39] Orlova VV van den Hil FE Petrus-Reurer S Drabsch Y Ten Dijke P Mummery CL. Generation, expansion and functional analysis of endothelial cells and pericytes derived from human pluripotent stem cells. Nat Protoc. (2014) 9:1514–31. 10.1038/nprot.2014.102 24874816

[40] Patsch C Challet-Meylan L Thoma EC Urich E Heckel T O'Sullivan JF. Generation of vascular endothelial and smooth muscle cells from human pluripotent stem cells. Nat Cell Biol. (2015) 17:994–1003. 10.1038/ncb3205 26214132 PMC4566857

[41] Tsujimoto H Kasahara T Sueta SI Araoka T Sakamoto S Okada C. A modular differentiation system maps multiple human kidney lineages from pluripotent stem cells. Cell Rep. (2020) 31:107476. 10.1016/j.celrep.2020.03.040 32268094

[42] Elcheva I Brok-Volchanskaya V Kumar A Liu P Lee JH Tong L. Direct induction of haematoendothelial programs in human pluripotent stem cells by transcriptional regulators. Nat Commun. (2014) 5:4372. 10.1038/ncomms5372 25019369 PMC4107340

[43] Wang K Lin RZ Hong X Ng AH Lee CN Neumeyer J. Robust differentiation of human pluripotent stem cells into endothelial cells *via* temporal modulation of ETV2 with modified mRNA. Sci Adv. (2020) 6:eaba7606. 10.1126/sciadv.aba7606 32832668 PMC7439318

[44] Srivastava D DeWitt N. *In vivo* cellular reprogramming: the next generation. Cell. (2016) 166:1386–96. 10.1016/j.cell.2016.08.055 27610565 PMC6234007

[45] Davis RL Weintraub H Lassar AB. Expression of a single transfected cDNA converts fibroblasts to myoblasts. Cell. (1987) 51:987–1000. 10.1016/0092-8674(87)90585-X 3690668

[46] Lee S Park C Han Ji W Kim Ju Y Cho K Kim Eun J. Direct reprogramming of human dermal fibroblasts into endothelial cells using ER71/ETV2. Circ Res. (2017) 120:848–61. 10.1161/CIRCRESAHA.116.309833 28003219 PMC5336520

[47] Morita R Suzuki M Kasahara H Shimizu N Shichita T Sekiya T. ETS transcription factor ETV2 directly converts human fibroblasts into functional endothelial cells. Proc Natl Acad Sci. (2015) 112:160. 10.1073/pnas.1413234112 25540418 PMC4291653

[48] Vestweber D, VE-cadherin: the major endothelial adhesion molecule controlling cellular junctions and blood vessel formation. Arterioscler Thromb Vasc Biol. (2008) 28:223–32. 10.1161/ATVBAHA.107.158014 18162609

[49] Starke RD Ferraro F Paschalaki KE Dryden NH McKinnon TA Sutton RE. Endothelial von Willebrand factor regulates angiogenesis. Blood. (2011) 117:1071–80. 10.1182/blood-2010-01-264507 21048155 PMC3035068

[50] Wong CW Wiedle G Ballestrem C Wehrle-Haller B Etteldorf S Bruckner M. PECAM-1/CD31 trans-homophilic binding at the intercellular junctions is independent of its cytoplasmic domain; evidence for heterophilic interaction with integrin alphavbeta3 in *Cis*. Mol Biol Cell. (2000) 11:3109–21. 10.1091/mbc.11.9.3109 10982404 PMC14979

[51] Lian X Bao X Al-Ahmad A Liu J Wu Y Dong W. Efficient differentiation of human pluripotent stem cells to endothelial progenitors *via* small-molecule activation of WNT signaling. Stem Cell Reports. (2014) 3:804–16. 10.1016/j.stemcr.2014.09.005 25418725 PMC4235141

[52] Voyta JC Via DP Butterfield CE Zetter BR. Identification and isolation of endothelial cells based on their increased uptake of acetylated-low density lipoprotein. J Cell Biol. (1984) 99:2034. 10.1083/jcb.99.6.2034 6501412 PMC2113570

[53] Zhang H Park Y Wu J Chen X Lee S Yang J. Role of TNF-alpha in vascular dysfunction. Clin Sci (Lond). (2009) 116:219–30. 10.1042/cs20080196 19118493 PMC2620341

[54] Li Z Hu S Ghosh Z Han Z Wu JC. Functional characterization and expression profiling of human induced pluripotent stem cell- and embryonic stem cell-derived endothelial cells. Stem Cells Dev. (2011) 20:1701–10. 10.1089/scd.2010.0426 21235328 PMC3182033

[55] Tiemeier GL Wang G Dumas SJ Sol W Avramut MC Karakach T. Closing the mitochondrial permeability transition pore in hiPSC-derived endothelial cells induces glycocalyx formation and functional maturation. Stem Cell Rep. (2019) 13:803–16. 10.1016/j.stemcr.2019.10.005 31680061 PMC6895683

[56] Jang S A Collin de l'Hortet Soto-Gutierrez A. Induced pluripotent stem cell-derived endothelial cells: overview, current advances, applications, and future directions. Am J Pathol. (2019) 189:502–12. 10.1016/j.ajpath.2018.12.004 30653953 PMC6902127

[57] Sharma A Sances S Workman MJ Svendsen CN. Multi-lineage human iPSC-derived platforms for disease modeling and drug discovery. Cell Stem Cell. (2020) 26:309–29. 10.1016/j.stem.2020.02.011 32142662 PMC7159985

[58] Bhatia SN Ingber DE. Microfluidic organs-on-chips. Nat Biotechnol. (2014) 32:760–72. 10.1038/nbt.2989 25093883

[59] Williams IM Wu JC. Generation of endothelial cells from human pluripotent stem cells. Arterioscler Thromb Vasc Biol. (2019) 39:1317–29. 10.1161/ATVBAHA.119.312265 31242035 PMC6597190

[60] Andrejecsk JW Hughes CCW. Engineering perfused microvascular networks into microphysiological systems platforms. Curr Opin Biomed Eng. (2018) 5:74–81. 10.1016/j.cobme.2018.02.002

[61] Natividad-Diaz SL Browne S Jha AK Ma Z Hossainy S Kurokawa YK. A combined hiPSC-derived endothelial cell and *in vitro* microfluidic platform for assessing biomaterial-based angiogenesis. Biomaterials. (2019) 194:73–83. 10.1016/j.biomaterials.2018.11.032 30583150 PMC6453535

[62] Crosby CO Valliappan D Shu D Kumar S Tu C Deng W. Quantifying the vasculogenic potential of induced pluripotent stem cell-derived endothelial progenitors in collagen hydrogels. Tissue Eng Part A. (2019) 25:746–58. 10.1089/ten.TEA.2018.0274 30618333 PMC6535961

[63] Maishi N Hida K. Tumor endothelial cells accelerate tumor metastasis. Cancer Sci. (2017) 108:1921–6. 10.1111/cas.13336 28763139 PMC5623747

[64] Bi Y Shirure VS Liu R Cunningham C Ding L Meacham JM. Tumor-on-a-chip platform to interrogate the role of macrophages in tumor progression. Integr Biol. (2020) 12:221–32. 10.1093/intbio/zyaa017 32930334 PMC7525664

[65] Zhao Z Nelson AR Betsholtz C Zlokovic BV. Establishment and dysfunction of the blood–brain barrier. Cell. (2015) 163:1064–1078. 10.1016/j.cell.2015.10.067 26590417 PMC4655822

[66] Stamatovic SM Keep RF Andjelkovic AV. Brain endothelial cell-cell junctions: how to “open” the blood brain barrier. Curr Neuropharmacol. (2008) 6:179–92. 10.2174/157015908785777210 19506719 PMC2687937

[67] Daneman R Prat A. The blood–brain barrier. Cold Spring Harb Perspect Biol. (2015) 7:a020412. 10.1101/cshperspect.a020412 25561720 PMC4292164

[68] Seo S Kim H Sung JH Choi N Lee K Kim HN. Microphysiological systems for recapitulating physiology and function of blood–brain barrier. Biomaterials. (2020) 232:119732. 10.1016/j.biomaterials.2019.119732 31901694

[69] Cucullo L Hossain M Puvenna V Marchi N Janigro D. The role of shear stress in blood–brain barrier endothelial physiology. BMC Neurosci. (2011) 12:40. 10.1186/1471-2202-12-40 21569296 PMC3103473

[70] Cecchelli R Berezowski V Lundquist S Culot M Renftel M Dehouck MP. Modelling of the blood–brain barrier in drug discovery and development. Nat Rev Drug Discov. (2007) 6:650–61. 10.1038/nrd2368 17667956

[71] Campisi M Shin Y Osaki T Hajal C Chiono V Kamm RD. 3D self-organized microvascular model of the human blood–brain barrier with endothelial cells, pericytes and astrocytes. Biomaterials. (2018) 180:117–29. 10.1016/j.biomaterials.2018.07.014 30032046 PMC6201194

[72] Park TE Mustafaoglu N Herland A Hasselkus R Mannix R FitzGerald EA. Hypoxia-enhanced Blood–Brain Barrier Chip recapitulates human barrier function and shuttling of drugs and antibodies. Nat Commun. (2019) 10:2621. 10.1038/s41467-019-10588-0 31197168 PMC6565686

[73] Lippmann ES Azarin SM Kay JE Nessler RA Wilson HK Al-Ahmad A. Derivation of blood–brain barrier endothelial cells from human pluripotent stem cells. Nat Biotechnol. (2012) 30:783–91. 10.1038/nbt.2247 22729031 PMC3467331

[74] Lippmann ES Al-Ahmad A Azarin SM Palecek SP Shusta EV. A retinoic acid-enhanced, multicellular human blood–brain barrier model derived from stem cell sources. Sci Rep. (2014) 4:4160. 10.1038/srep04160 24561821 PMC3932448

[75] Vatine GD Barrile R Workman MJ Sances S Barriga BK Rahnama M. Human iPSC-derived Blood–Brain Barrier Chips enable disease modeling and personalized medicine applications. Cell Stem Cell. (2019) 24:995–1005 e6. 10.1016/j.stem.2019.05.011 31173718

[76] Faley SL Neal EH Wang JX Bosworth AM Weber CM Balotin KM. iPSC-derived brain endothelium exhibits stable, long-term barrier function in perfused hydrogel scaffolds. Stem Cell Rep. (2019) 12:474–87. 10.1016/j.stemcr.2019.01.009 30773484 PMC6409430

[77] Workman MJ Svendsen CN. Recent advances in human iPSC-derived models of the blood–brain barrier. Fluids Barriers CNS. (2020) 17:30. 10.1186/s12987-020-00191-7 32321511 PMC7178976

[78] Adriani G Ma D Pavesi A Kamm RD Goh EL. A 3D neurovascular microfluidic model consisting of neurons, astrocytes and cerebral endothelial cells as a blood–brain barrier. Lab Chip. (2017) 17:448–59. 10.1039/c6lc00638h 28001148

[79] Bang S Lee SR Ko J Son K Tahk D Ahn J. A low permeability microfluidic blood–brain barrier platform with direct contact between perfusable vascular network and astrocytes. Sci Rep. (2017) 7:8083. 10.1038/s41598-017-07416-0 28808270 PMC5556097

[80] Takebe T Sekine K Enomura M Koike H Kimura M Ogaeri T. Vascularized and functional human liver from an iPSC-derived organ bud transplant. Nature. (2013) 499:481–4. 10.1038/nature12271 23823721

[81] Asai A Aihara E Watson C Mourya R Mizuochi T Shivakumar P. Paracrine signals regulate human liver organoid maturation from induced pluripotent stem cells. Development. (2017) 144:1056–64. 10.1242/dev.142794 28275009 PMC5358109

[82] Camp JG Sekine K Gerber T Loeffler-Wirth H Binder H Gac M. Multilineage communication regulates human liver bud development from pluripotency. Nature. (2017) 546:533–8. 10.1038/nature22796 28614297

[83] Danoy M Poulain S Koui Y Tauran Y Scheidecker B Kido T. Transcriptome profiling of hiPSC-derived LSECs with nanoCAGE. Mol Omics. (2020) 16:138–46. 10.1039/c9mo00135b 31989141

[84] de Haan W Oie C Benkheil M Dheedene W Vinckier S Coppiello G. Unraveling the transcriptional determinants of liver sinusoidal endothelial cell specialization. Am J Physiol Gastrointest Liver Physiol. (2020) 318: G803–15. 10.1152/ajpgi.00215.2019 32116021 PMC7191457

[85] Gage BK Liu JC Innes BT MacParland SA McGilvray ID Bader GD. Generation of functional liver sinusoidal endothelial cells from human pluripotent stem-cell-derived venous angioblasts. Cell Stem Cell. (2020) 27:254–69.e9. 10.1016/j.stem.2020.06.007 32640183

[86] Lucendo-Villarin B Meseguer-Ripolles J Drew J Fischer L Ma E Flint O. Development of a cost-effective automated platform to produce human liver spheroids for basic and applied research. Biofabrication. (2020) 13:015009 10.1088/1758-5090/abbdb2 33007774

[87] Serluca FC Drummond IA Fishman MC. Endothelial signaling in kidney morphogenesis: a role for hemodynamic forces. Curr Biol. (2002) 12:492–7. 10.1016/s0960-9822(02)00694-2 11909536

[88] Nishinakamura R, Human kidney organoids: progress and remaining challenges. Nat Rev Nephrol. (2019) 15:613–24. 10.1038/s41581-019-0176-x 31383997

[89] Takasato M Wymeersch FJ. Challenges to future regenerative applications using kidney organoids. Curr Opin Biomed Eng. (2020) 13:144–51. 10.1016/j.cobme.2020.03.00333034708

[90] Homan KA Gupta N Kroll KT Kolesky DB Skylar-Scott M Miyoshi T. Flow-enhanced vascularization and maturation of kidney organoids *in vitro*. Nat Methods. (2019) 16:255–62. 10.1038/s41592-019-0325-y 30742039 PMC6488032

[91] Sharmin S Taguchi A Kaku Y Yoshimura Y Ohmori T Sakuma T. Human induced pluripotent stem cell–derived podocytes mature into vascularized glomeruli upon experimental transplantation. J Am Soc Nephrol. (2016) 27:1778. 10.1681/ASN.2015010096 26586691 PMC4884101

[92] van den Berg CW Ritsma L Avramut MC Wiersma LE van den Berg BM Leuning DG. Renal subcapsular transplantation of PSC-derived kidney organoids induces neo-vasculogenesis and significant glomerular and tubular maturation *in vivo*. Stem Cell Rep. (2018) 10:751–65. 10.1016/j.stemcr.2018.01.041 29503086 PMC5918682

[93] Tanigawa S Islam M Sharmin S Naganuma H Yoshimura Y Haque F. Organoids from nephrotic disease-derived iPSCs identify impaired NEPHRIN localization and slit diaphragm formation in kidney podocytes. Stem Cell Rep. (2018) 11:727–40. 10.1016/j.stemcr.2018.08.003 30174315 PMC6135868

[94] Bantounas I Ranjzad P Tengku F Silajdzic E Forster D Asselin MC. Generation of functioning nephrons by implanting human pluripotent stem cell-derived kidney progenitors. Stem Cell Rep. (2018) 10:766–79. 10.1016/j.stemcr.2018.01.008 29429961 PMC5918196

[95] Rayner SG Phong KT Xue J Lih D Shankland SJ Kelly EJ. Reconstructing the human renal vascular–tubular unit *in vitro*. Adv Healthc Mater. (2018) 7:e1801120. 10.1002/adhm.201801120 30379416 PMC6478624

[96] Homan KA Kolesky DB Skylar-Scott MA Herrmann J Obuobi H Moisan A. Bioprinting of 3D convoluted renal proximal tubules on perfusable chips. Sci Rep. (2016) 6:34845. 10.1038/srep34845 27725720 PMC5057112

[97] Vedula EM Alonso JL Arnaout MA Charest JL. A microfluidic renal proximal tubule with active reabsorptive function. PLoS ONE. (2017) 12: e0184330. 10.1371/journal.pone.0184330 29020011 PMC5636065

[98] Lin NYC Homan KA Robinson SS Kolesky DB Duarte N Moisan A. Renal reabsorption in 3D vascularized proximal tubule models. Proc Natl Acad Sci USA. (2019) 116:5399–404. 10.1073/pnas.1815208116 30833403 PMC6431199

[99] Bai J Wang C. Organoids and microphysiological systems: new tools for ophthalmic drug discovery. Front Pharmacol. (2020) 11:407. 10.3389/fphar.2020.00407 32317971 PMC7147294

[100] Wimmer RA Leopoldi A Aichinger M Kerjaschki D Penninger JM. Generation of blood vessel organoids from human pluripotent stem cells. Nat Protoc. (2019) 14:3082–100. 10.1038/s41596-019-0213-z 31554955

[101] Morizane R, Modelling diabetic vasculopathy with human vessel organoids. Nat Rev Nephrol. (2019) 15:258–60. 10.1038/s41581-019-0125-8 30778152 PMC6605777

[102] Wimmer RA Leopoldi A Aichinger M Wick N Hantusch B Novatchkova M. Human blood vessel organoids as a model of diabetic vasculopathy. Nature. (2019) 565:505–10. 10.1038/s41586-018-0858-8 30651639 PMC7116578

[103] Ronaldson-Bouchard K Vunjak-Novakovic G. Organs-on-a-chip: a fast track for engineered human tissues in drug development. Cell Stem Cell. (2018) 22:310–24. 10.1016/j.stem.2018.02.011 29499151 PMC5837068

[104] West JD Austin ED Gaskill C Marriott S Baskir R Bilousova G. Identification of a common Wnt-associated genetic signature across multiple cell types in pulmonary arterial hypertension. Am J Physiol Cell Physiol. (2014) 307:C415–30. 10.1152/ajpcell.00057.2014 24871858 PMC4154073

[105] Gu M Shao NY Sa S Li D Termglinchan V Ameen M. Patient-specific iPSC-derived endothelial cells uncover pathways that protect against pulmonary hypertension in BMPR2 mutation carriers. Cell Stem Cell. (2017) 20:490–504.e5. 10.1016/j.stem.2016.08.019 28017794 PMC5500296

[106] Hitomi T Habu T Kobayashi H Okuda H Harada KH Osafune K. Downregulation of securin by the variant RNF213 R4810K (rs112735431, G>A) reduces angiogenic activity of induced pluripotent stem cell-derived vascular endothelial cells from moyamoya patients. Biochem Biophys Res Commun. (2013) 438:13–9. 10.1016/j.bbrc.2013.07.004 23850618

[107] Lim RG Quan C Reyes-Ortiz AM Lutz SE Kedaigle AJ Gipson TA. Huntington's disease iPSC-derived brain microvascular endothelial cells reveal WNT-mediated angiogenic and blood–brain barrier deficits. Cell Rep. (2017) 19:1365–77. 10.1016/j.celrep.2017.04.021 28514657 PMC5646270

[108] Atchison L Abutaleb NO Snyder-Mounts E Gete Y Ladha A Ribar T. iPSC-derived endothelial cells affect vascular function in a tissue-engineered blood vessel model of Hutchinson–Gilford progeria syndrome. Stem Cell Rep. (2020) 14:325–37. 10.1016/j.stemcr.2020.01.005 32032552 PMC7013250

[109] Kelleher J Dickinson A Cain S Hu Y Bates N Harvey A. Patient-specific iPSC model of a genetic vascular dementia syndrome reveals failure of mural cells to stabilize capillary structures. Stem Cell Reports. (2019) 13:817–31. 10.1016/j.stemcr.2019.10.004 31680059 PMC6893064

[110] Ong SB Lee WH Shao NY Ismail NI Katwadi K Lim MM. Calpain inhibition restores autophagy and prevents mitochondrial fragmentation in a human iPSC model of diabetic endotheliopathy. Stem Cell Rep. (2019) 12:597–610. 10.1016/j.stemcr.2019.01.017 30799273 PMC6411483

[111] Oikari LE Pandit R Stewart R Cuni-Lopez C Quek H Sutharsan R. Altered brain endothelial cell phenotype from a familial Alzheimer mutation and its potential implications for amyloid clearance and drug delivery. Stem Cell Rep. (2020) 14:924–39. 10.1016/j.stemcr.2020.03.011 32275861 PMC7220857

[112] DiMasi JA Grabowski HG Hansen RW. Innovation in the pharmaceutical industry: new estimates of R&D costs. J Health Econ. (2016) 47:20–33. 10.1016/j.jhealeco.2016.01.012 26928437

[113] Cochrane A Albers HJ Passier R Mummery CL van den Berg A Orlova VV. Advanced *in vitro* models of vascular biology: human induced pluripotent stem cells and organ-on-chip technology. Adv Drug Deliv Rev. (2019) 140:68–77. 10.1016/j.addr.2018.06.007 29944904

[114] Adams WJ Zhang Y Cloutier J Kuchimanchi P Newton G Sehrawat S. Functional vascular endothelium derived from human induced pluripotent stem cells. Stem Cell Rep. (2013) 1:105–13. 10.1016/j.stemcr.2013.06.007 24052946 PMC3757754

[115] Sharma A Burridge PW McKeithan WL Serrano R Shukla P Sayed N. High-throughput screening of tyrosine kinase inhibitor cardiotoxicity with human induced pluripotent stem cells. Sci Transl Med. (2017) 9:eaaf2584. 10.1126/scitranslmed.aaf2584 28202772 PMC5409837

[116] Vazao H Rosa S Barata T Costa R Pitrez PR Honorio I. High-throughput identification of small molecules that affect human embryonic vascular development. Proc Natl Acad Sci USA. (2017) 114: E3022–31. 10.1073/pnas.1617451114 28348206 PMC5393190

[117] Weng KC Kurokawa YK Hajek BS Paladin JA Shirure VS George SC. Human induced pluripotent stem-cardiac-endothelial-tumor-on-a-chip to assess anticancer efficacy and cardiotoxicity. Tissue Eng Part C Methods. (2020) 26:44–55. 10.1089/ten.TEC.2019.0248 31797733 PMC6983745

[118] Monteil V Kwon H Prado P Hagelkruys A Wimmer RA Stahl M. Inhibition of SARS-CoV-2 infections in engineered human tissues using clinical-grade soluble human ACE2. Cell. (2020) 181:905–13.e7. 10.1016/j.cell.2020.04.004 32333836 PMC7181998

[119] Bezenah JR Kong YP Putnam AJ. Evaluating the potential of endothelial cells derived from human induced pluripotent stem cells to form microvascular networks in 3D cultures. Sci Rep. (2018) 8:2671. 10.1038/s41598-018-20966-1 29422650 PMC5805762

[120] Rosa S Praca C Pitrez PR Gouveia PJ Aranguren XL Ricotti L. Functional characterization of iPSC-derived arterial- and venous-like endothelial cells. Sci Rep. (2019) 9:3826. 10.1038/s41598-019-40417-9 30846769 PMC6405900

[121] Clayton ZE Tan RP Miravet MM Lennartsson K Cooke JP Bursill CA. Induced pluripotent stem cell-derived endothelial cells promote angiogenesis and accelerate wound closure in a murine excisional wound healing model. Biosci Rep. (2018) 38:BSR20180563. 10.1042/BSR20180563 29976773 PMC6066657

[122] Foster AA Dewi RE Cai L Hou L Strassberg Z Alcazar CA. Protein-engineered hydrogels enhance the survival of induced pluripotent stem cell-derived endothelial cells for treatment of peripheral arterial disease. Biomater Sci. (2018) 6:614–22. 10.1039/c7bm00883j 29406542 PMC5829050

[123] Tan RP Chan AHP Lennartsson K Miravet MM Lee BSL Rnjak-Kovacina J. Integration of induced pluripotent stem cell-derived endothelial cells with polycaprolactone/gelatin-based electrospun scaffolds for enhanced therapeutic angiogenesis. Stem Cell Res Ther. (2018) 9:70. 10.1186/s13287-018-0824-2 29562916 PMC5863387

[124] Song HHG Rumma RT Ozaki CK Edelman ER Chen CS. Vascular tissue engineering: progress, challenges, and clinical promise. Cell Stem Cell. (2018) 22:340–54. 10.1016/j.stem.2018.02.009 29499152 PMC5849079

[125] Takebe T Zhang R Koike H Kimura M Yoshizawa E Enomura M. Generation of a vascularized and functional human liver from an iPSC-derived organ bud transplant. Nat Protoc. (2014) 9:396–409. 10.1038/nprot.2014.020 24457331

[126] Abaci HE Guo Z Coffman A Gillette B Lee WH Sia SK. Human skin constructs with spatially controlled vasculature using primary and iPSC-derived endothelial cells. Adv Healthc Mater. (2016) 5:1800–7. 10.1002/adhm.201500936 27333469 PMC5031081

[127] Zhang B Montgomery M Chamberlain MD Ogawa S Korolj A Pahnke A. Biodegradable scaffold with built-in vasculature for organ-on-a-chip engineering and direct surgical anastomosis. Nat Mater. (2016) 15:669–78. 10.1038/nmat4570 26950595 PMC4879054

[128] Chang WG Niklason LE. A short discourse on vascular tissue engineering. NPJ Regen Med. (2017) 2:7. 10.1038/s41536-017-0011-6 29057097 PMC5649630

[129] Nakayama KH Joshi PA Lai ES Gujar P Joubert LM Chen B. Bilayered vascular graft derived from human induced pluripotent stem cells with biomimetic structure and function. Regen Med. (2015) 10:745–55. 10.2217/rme.15.45 26440211 PMC4760352

[130] Krawiec JT Weinbaum JS Liao HT Ramaswamy AK Pezzone DJ Josowitz AD. *In vivo* functional evaluation of tissue-engineered vascular grafts fabricated using human adipose-derived stem cells from high cardiovascular risk populations. Tissue Eng Part A. (2016) 22:765–75. 10.1089/ten.TEA.2015.0379 27079751 PMC4876541

[131] Krawiec JT Liao H-T Kwan L D'Amore A Weinbaum JS Rubin JP. Evaluation of the stromal vascular fraction of adipose tissue as the basis for a stem cell-based tissue-engineered vascular graft. J Vasc Surg. (2017) 66:883–90.e1. 10.1016/j.jvs.2016.09.034 28017585 PMC5481505

